# Virus-Induced Gene Silencing-Based Functional Analyses Revealed the Involvement of Several Putative Trehalose-6-Phosphate Synthase/Phosphatase Genes in Disease Resistance against *Botrytis cinerea* and *Pseudomonas syringae* pv. *tomato* DC3000 in Tomato

**DOI:** 10.3389/fpls.2016.01176

**Published:** 2016-08-04

**Authors:** Huijuan Zhang, Yongbo Hong, Lei Huang, Shixia Liu, Limei Tian, Yi Dai, Zhongye Cao, Lihong Huang, Dayong Li, Fengming Song

**Affiliations:** ^1^College of Life Science, Taizhou UniversityTaizhou, China; ^2^National Key Laboratory for Rice Biology, Institute of Biotechnology, Zhejiang UniversityHangzhou, China

**Keywords:** Trehalose, trehalose-6-phosphate synthase, trehalose-6-phosphate phosphatase (TPP), *Botrytis cinerea*, *Pseudomonas syringae* pv. *tomato* DC3000, disease resistance, defense response

## Abstract

Trehalose and its metabolism have been demonstrated to play important roles in control of plant growth, development, and stress responses. However, direct genetic evidence supporting the functions of trehalose and its metabolism in defense response against pathogens is lacking. In the present study, genome-wide characterization of putative trehalose-related genes identified 11 *SlTPSs* for trehalose-6-phosphate synthase, 8 *SlTPPs* for trehalose-6-phosphate phosphatase and one *SlTRE1* for trehalase in tomato genome. Nine *SlTPSs*, 4 *SlTPPs*, and *SlTRE1* were selected for functional analyses to explore their involvement in tomato disease resistance. Some selected *SlTPSs*, *SlTPPs*, and *SlTRE1* responded with distinct expression induction patterns to *Botrytis cinerea* and *Pseudomonas syringae* pv. *tomato* (*Pst*) DC3000 as well as to defense signaling hormones (e.g., salicylic acid, jasmonic acid, and a precursor of ethylene). Virus-induced gene silencing-mediated silencing of *SlTPS3*, *SlTPS4*, or *SlTPS7* led to deregulation of ROS accumulation and attenuated the expression of defense-related genes upon pathogen infection and thus deteriorated the resistance against *B. cinerea* or *Pst* DC3000. By contrast, silencing of *SlTPS5* or *SlTPP2* led to an increased expression of the defense-related genes upon pathogen infection and conferred an increased resistance against *Pst* DC3000. Silencing of *SlTPS3*, *SlTPS4*, *SlTPS5*, *SlTPS7*, or *SlTPP2* affected trehalose level in tomato plants with or without infection of *B. cinerea* or *Pst* DC3000. These results demonstrate that *SlTPS3*, *SlTPS4*, *SlTPS5*, *SlTPS7*, and *SlTPP2* play roles in resistance against *B. cinerea* and *Pst* DC3000, implying the importance of trehalose and tis metabolism in regulation of defense response against pathogens in tomato.

## Introduction

Trehalose (α-D-glucopyranosyl α-D-glucopyranoside) is a ubiquitously distributed non-reducing disaccharide (Elbein et al., 2003). The biosynthesis and degradation of trehalose in plants include three consecutive enzymatic steps. Firstly, trehalose-6-phosphate synthase (TPS) catalyzes the synthesis of trehalose-6-phosphate (T6P), which is subsequently dephosphorylated into trehalose by T-6-phosphate phosphatase (TPP). Furthermore, the synthesized trehalose can be hydrolyzed into two glucose monomers by the enzyme trehalase (TRE) (Schluepmann and Paulb, 2009). Biochemically, trehalose has been shown to be capable of stabilizing proteins and lipid membranes in cells and the trehalose metabolism is essentially required for some general metabolic pathways such as sugar status, carbon assimilation, biosynthesis, and degradation of starch in plants (Goddijn and van Dun, 1999; Paul et al., 2008; Lunn et al., 2014).

The TPSs and TPPs constitute two multi-gene families while the TRE is present as a single-copy gene in most of sequenced plant genomes (Lunn, 2007). For example, *Arabidopsis* contains 11 TPS genes (AtTPS1–AtTPS11) and 10 TPP genes (AtTPPA–AtTPPJ) (Leyman et al., 2001; Vandesteene et al., 2012) while rice has 11 TPS (OsTPS1–OsTPS11) and 11 TPP (OsTPP1–OsTPP11) (Ge et al., 2008; Zhang et al., 2011). Similar numbers of TPS and/or TPP genes were identified in wheat (Xie et al., 2015), maize (Henry et al., 2014; Zhou et al., 2014), poplar (Yang et al., 2012), and cotton (Mu et al., 2016). Plant TPSs can be divided into two groups with differences in structural features and biochemical activity. Group I TPSs contain both TPS and TPP domains and the *Arabidopsis* AtTPS1, AtTPS2, and AtTPS4 are active enzymes (Blazquez et al., 1998; Vandesteene et al., 2010; Delorge et al., 2015). Group II TPSs contain both TPS and TPP domains and most of them harbor conserved phosphatase domains (Vandesteene et al., 2010; Zhang et al., 2011). Whereas most of the *Arabidopsis* Class II TPSs are not active enzymes (Ramon et al., 2009), AtTPS6 and AtTPS11 were found to possess TPS or TPP activity (Chary et al., 2008; Singh et al., 2011). In addition, it was shown that the OsTPSs can form TPS complexes, which may potentially regulate T6P levels in plants (Zhang et al., 2011). By contrast, plant TPPs contain unique TPP domains with conserved phosphatase domains and all of them possess TPP activities (Shima et al., 2007).

Extensive genetic studies using loss-of-function and gain-of-function mutants have demonstrated that the trehalose metabolism plays critical roles in control of plant growth and development including embryo development, leaf morphology and senescence, and flowering (Satoh-Nagasawa et al., 2006; Gómez et al., 2010; Wingler et al., 2012; Nunes et al., 2013; Wahl et al., 2013) (for reviews, see Ramon and Rolland, 2007; Paul et al., 2008; Ponnu et al., 2011; Lunn et al., 2014; Tsai and Gazzarrini, 2014). Increasing evidence also supports that trehalose and its metabolism function in plant response to a number of unfavorable environmental conditions such as extreme temperatures, drought, salt and oxidative stresses (Iordachescu and Imai, 2008; Fernandez et al., 2012; Delorge et al., 2014; Lunn et al., 2014; Figueroa et al., 2016). For example, mutations in *Arabidopsis AtTPS5* and *AtTPPD* impaired the tolerance to extreme temperatures and salt stress, respectively (Suzuki et al., 2008; Krasensky et al., 2014; Wang et al., 2016). By contrast, overexpression of *AtTRE1* in *Arabidopsis*, *OsTPS1* and *OsTPP1* in rice, and heterologous *TPS* and *TPP* genes in transgenic plants confer improved abiotic stress tolerance (Garg et al., 2002; Jang et al., 2003; Pramanik and Imai, 2005; Karim et al., 2007; Miranda et al., 2007; Ge et al., 2008; Debast et al., 2011; Li et al., 2011; Van Houtte et al., 2013a). Thus, modulation of the endogenous trehalose metabolism is a promising strategy to improve stress tolerance in crop plants (Lunn et al., 2014).

There is also emerging evidence indicating that trehalose and its metabolism are involved in plant responses to biotic factors such as pathogenic microorganisms and herbivorous insects (Lunn et al., 2014). It was shown that exogenous trehalose acts as an elicitor of plant defense response (Bae et al., 2005) and can induce resistance in wheat plants against powdery mildew disease (Reignault et al., 2001; Renard-Merlier et al., 2007; Tayeh et al., 2014). Treatment with an inhibitor of trehalase, validamycin A, induced resistance to *Fusarium* wilt and late blight diseases, although exogenous trehalose did not confer resistance to powdery mildew disease (Ishikawa et al., 2005). Furthermore, expression of *AtTPS11* and *AtTRE* in *Arabidopsis* plants was induced by infection with *Tobacco mosaic virus* (Golem and Culver, 2003) or *Plasmodiophora brassica* (Brodmann et al., 2002). Excess levels of trehalose accumulated in *Arabidopsis* roots after infection with a pathogenic nematode (Hofmann et al., 2010) or in citrus leaves infected with *Xanthomonas citri* subsp. *citri* (Piazza et al., 2015). Most recently, it was found that a *Ralstonia solanacearum* type III effector, ripTPS, is a functional TPS enzyme that elicits a hypersensitive response on tobacco (Poueymiro et al., 2014). However, genetic evidence originated from disease phenotype analysis of loss-of-function or gain-of-function mutants or transgenic lines is lacking to support the function of trehalose metabolism in pathogen resistance in plants. On the other hand, exogenous trehalose can also serve as a potential sign of dangers from infestation of herbivorous insects. For example, infestation of *Arabidopsis* and tomato plants by peach potato aphid led to accumulation of trehalose (Singh and Shah, 2012; Hodge et al., 2013) and mutation in *Arabidopsis AtTPS11* impaired both the trehalose accumulation and resistance against aphids, suggesting that treahlose is an essential signal in the defense process (Singh et al., 2011).

The present study was aimed to explore the involvement of the trehalose metabolism in disease resistance against *Botrytis cinerea*, a necrotrophic fungal pathogen, and *Pseudomonas syringae* pv. *tomato* DC3000, a (hemi)biotrphic bacterial pathogen, in tomato. We identified 11 *SlTPS*, 8 *SlTPP*, and one *SlTRE* genes in tomato genome. Virus-induced gene silencing (VIGS)-based functional analyses revealed that VIGS-mediated silencing of *SlTPS3*, *SlTPS4*, or *SlTPS7* deteriorated the resistance against *B. cinerea* and *Pst* DC3000, whereas silencing of *SlTPS5* or *SlTPP2* conferred an increased resistance against *Pst* DC3000. These findings demonstrate the importance of trehalose and its metabolic genes in regulation of defense response against pathogens in tomato.

## Materials and Methods

### Plant Growth and Treatments

Tomato (*Solanum lycopersicum*) cv. Suhong2003 was used for most of the experiments except that cultivar MicroTom was used in whole plant inoculation assays with *B. cinerea*. Growth of tomato plants and treatment with hormones were the same as previously described (Li et al., 2014b). Leaf samples were harvested at specific time points and stored at -80°C until use.

### Characterization of *SlTPS, SlTPP*, and *SlTRE1* Genes

Tomato genome database at the SOL Genomics Network^1^ (SGN) was searched using BlastP program with *Arabidopsis* AtTPSs, AtTPPs, and AtTRE1 as queries and the predicted nucleotide and amino acid sequences for *SlTPSs*, *SlTPPs*, and *SlTRE1* were downloaded. Conserved TPS and TPP domains in the predicted SlTPS and SlTPP proteins were analyzed using the Conserved Domain Search program at NCBI website^2^ under default parameters and the Motif Scan program at MyHits website^3^ with the following parameters (hamap, pfam_fs, and pfam_Is). Putative ESTs or UniGenes and full-length cDNAs were searched against the tomato genome database and NCBI GenBank database, respectively, using predicted nucleotide sequences as queries. Phylogenetic trees for tomato, *Arabidopsis* and rice TPSs and TPPs were constructed using the neighbor-joining method of the MEGA6 program with the *p*-distance and complete deletion option parameters using a bootstrapping method with 1000 replicates.

### VIGS Vector Construction and Agroinfiltration

Fragments of 300–400 bp, spanning partial 5′-UTR and coding sequences (**Supplementary file 1**), for selected *SlTPSs*, *SlTPPs*, and *SlTRE1* were amplified by PCR with respective pairs of gene-specific primers (**Supplementary Table S1**). The amplified PCR products were digested with corresponding restriction enzymes (*Xba*I/*Xho*I or *Eco*RI/*Bam*HI) and cloned into TRV2, yielding recombinant plasmids TRV-SlTPSs, TRV-SlTPPs, and TRV-SlTRE1. After confirmation by sequencing, the correct recombinant plasmids were transformed into *Agrobacterium tumefaciens* strain GV3101 by electroporation and positive clones were selected by colony PCR. Cultivation of agrobacteria carrying different constructs of TRV-SlTPSs, TRV-SlTPPs and TRV-SlTRE1 and agroinfiltration for standard VIGS were carried out as described before (Li et al., 2014b). In all VIGS assays, a construct of TRV-PDS (*Phytoene desaturase*) was included as positive controls for silencing evaluation of the VIGS procedure (Liu et al., 2002).

### Pathogen Inoculation and Disease Assays

Inoculation of tomato plants with *B. cinerea* was carried out using two different methods as described previously (Li et al., 2014b). Spore concentration in the inoculum was adjusted to 1 × 10^5^ spores/mL. In detached leaf disease assays, leaves were collected from the second and third branches of 4-week-old plants and placed on wet cheesecloth in trays. After inoculation by dropping 5 μL of spore suspension on the surface of the detached leaves, the trays were covered with transparent plastic films to maintain high humidity. Lesion sizes were measured 4 days later. In whole plant disease assays, spore suspension was sprayed evenly on leaf surface of 4-week-old plants, which were then kept in high humidity in the growth room. Photos were taken at 4 days after inoculation. The inoculated leaves were harvested for gene expression and the determination of *in planta* fungal growth (Li et al., 2014b).

Plant inoculation with *Pst* DC3000 was carried out following previously described method (Li et al., 2014b). Briefly, plants were submerged into bacterial suspension (OD_600_ = 0.0002 in 10 mM MgCl_2_ with 0.04% Silwet l–77) and vacuum infiltrated under a -40 Kpa pressure for 1.5 min using a vacuum pump. The inoculated plants were kept in the growth room for growth with high humidity. Measurement of *in planta* bacterial growth was done as before (Li et al., 2014b).

### RNA Extraction and qRT-PCR

Frozen leaf samples were homogenized in liquid nitrogen using a mortar and pestle. Total RNA was extracted using Trizol reagent (Invitrogen, Shanghai, China). First-strand cDNAs were synthesized using PrimeScript RT regent kit (TaKaRa, Dalian, China) and used for amplification of VIGS fragments and qRT-PCR analyses of gene expression. qRT-PCR was done with SYBR Premix Ex Taq (TaKaRa, Dalian, China) on a CFX96 real-time PCR detection system (Bio-Rad, Hercules, CA, USA) and the conditions consisted of 40 cycles of denaturation at 95°C for 15 s, annealing at 55 or 60°C for 15 s and an extension at 72°C for 15 s. Dissociation curves were generated at the end of the PCR cycle to verify that a single product was amplified in the PCR reactions for each of the target genes using the software provided with the Bio-Rad System. Transcript levels of the target genes were normalized with the transcript level of a tomato *Actin* gene. Relative expression was calculated using 2^-ΔΔCT^ method as described previously. Gene-specific primers used in qRT-PCR are listed in **Supplementary Table S1**.

### Measurement of Trehalose Content

Measurement of trehalose content in tomato leaves was performed according to a previously described method (Jang et al., 2003; Ge et al., 2008). Briefly, leaf samples (2 g) were ground in liquid nitrogen and extracted in 20 ml boiling water for 10 min. The extract was centrifuged at 12,500*g* for 10 min and the supernatant was passed through a 0.45 μm filter. Trehalose content was determined by high-performance ion chromatography (DX500 HPIC system, Dionex 500, CA, USA). Commercial trehalose (Sigma, MO, USA) was used as a standard to calculate trehalose content in samples.

### Detection of H_2_O_2_

Leaves collected at 0 and 24 h from *B. cinerea*-inoculated plants or at 0 and 48 h from *Pst* DC3000-inoculatd plants were used for detection of H_2_O_2_ accumulation by DAB staining as described before (Li et al., 2014b; Liu et al., 2014). Accumulation of H_2_O_2_ in stained leaves was visualized using a digital camera.

### Experiment Design and Data Analysis

All experiments were independently repeated three times and three replicates were included in each of the independent experiments. At least 10 plants were used in each of independent experiments in whole plant inoculation assays with *B. cinerea* or with *Pst* DC3000 or leaves from 10 individual plants were collected for detached leaf inoculation assays with *B. cinerea*. Leaf samples were collected from three individual plants for analyses of H_2_O_2_ accumulation, trehalose content and gene expression. Data from three independent experiments were statistically analyzed according to the Student’s *t*-test and the probability of *p* < 0.05 was considered as significant difference.

## Results

### Characterization of *SlTPS*, *SlTPP*, and *SlTRE1* Genes in Tomato

By Blastp searches against the tomato genome database using the characterized *Arabidopsis* AtTPSs, AtTPPs, and AtTRE1 as queries, we identified 11, 8, and 1 loci in tomato genome that were predicted to encode TPS, TPP, and TRE and designated as *SlTPS1-11*, *SlTPP1-8*, and *SlTRE1*, respectively (**Table 1**), based on their chromosomal locations.

**Table 1:** Information on the *SlTPS*, *SlTPP*, and *SlTRE* genes and proteins.

Family	Genes	Loci in SOL	Accessions in GenBank	ORF (bp)	Protein size and domains			UniGenes in SOL/cDNAs in GenBank
						
					size (aa)	TPS	TPP	
TPS	SlTPS1	Solyc01g005210	XP_004228746	2574	857	Yes	Yes	SGN-U574042, SGN-U600459, SGN-U574043
	SlTPS2	Solyc02g071590	XP_010316884	2832	943	Yes	Yes	–
	SlTPS3	Solyc02g072150	XP_004233035	2556	851	Yes	Yes	SGN-U575044, SGN-U575051, SGN-U575049
	SlTPS4	Solyc04g025940	XP_004237260	2574	857	Yes	Yes	SGN-U576714, SGN-U567013
	SlTPS5	Solyc05g005750	XP_004238680	2556	851	Yes	Yes	SGN-U576715
	SlTPS6	Solyc07g006500	XP_010323144	2631	876	Yes	Yes	SGN-U576716, AB368491
	SlTPS7	Solyc07g055300	XP_004243268	2577	858	Yes	Yes	SGN-U585228, SGN-U599997
	SlTPS8	Solyc07g062140	NP_001234879	2781	926	Yes	Yes	SGN-U579539, SGN-U580026, EF151131
	SlTPS9	Solyc08g076650	XP_004245918	2589	862	Yes	Yes	SGN-U583981
	SlTPS10	Solyc10g007950	XP_004248198	2574	857	Yes	Yes	SGN-U584220, SGN-U600516
	SlTPS11^a^	Solyc10g046770	XM_010329326	735	244	–	Yes	–
TPP	SlTPP1	Solyc03g007290	XP_004234173	1011	336	No	Yes	–
	SlTPP2	Solyc03g083960	XP_010317997	1104	367	No	Yes	SGN-U584704, AK319855, AK247068, AK322638
	SlTPP3	Solyc04g054930	XP_004237406	1167	388	No	Yes	SGN-U570949, AK320358
	SlTPP4	Solyc04g072920	XP_004237894	1098	365	No	Yes	SGN-U575865, AK321917
	SlTPP5	Solyc04g082550	XP_004238632	882	293	No	Yes	–
	SlTPP6	Solyc05g051880	XP_010321465	1047	348	No	Yes	–
	SlTPP7	Solyc06g060600	XP_004242008	1020	339	No	Yes	–
	SlTPP8	Solyc08g079060	XP_004245739	1161	386	No	Yes	SGN-U584816, SGN-U584817, SGN-U568331
TRE	SlTRE1	Solyc08g082860	XP_004245478	1746	581	–	–	SGN-U568010, AK320041


*^a^the predicted ORFs seems incomplete for intact proteins.*

**FIGURE 1:**
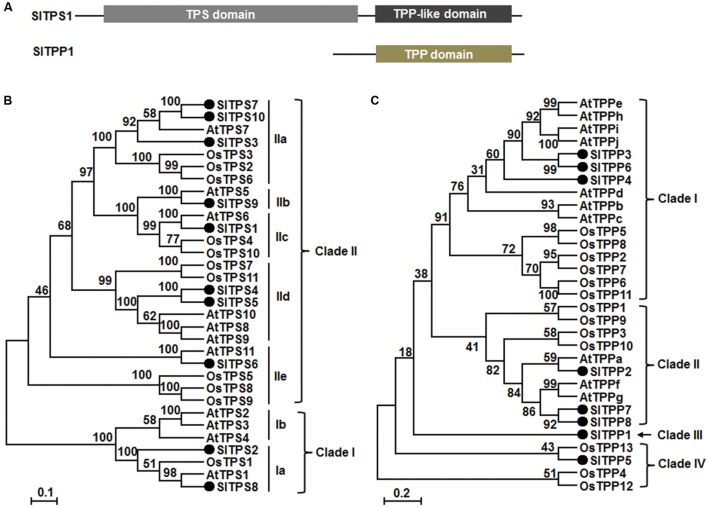
**Structural features and phylogenetic tree of SlTPSs and SlTPPs with *Arabidopsis* and rice TPSs and TPPs.** **(A)** Structures of SlTPS1 and SlTPP1. Conserved domains are indicated. **(B,C)** Phylogenetic tree of SlTPSs and SlTPPs. Phylogenetic trees were constructed by neighbor-joining method using MEGA program version 6.05. SlTPSs and SlTPPs in the trees are indicated by filled circles and different clades are labeled at right of the trees.

Among the 11 predicted SlTPSs, SlTPS1–SlTPS10 are complete TPSs containing both TPS and TPP-like domains (**Figure 1A**), but the predicted SlTPS11 is an incomplete TPS that only contains a partial TPP domain (**Table 1**). Nine of 11 *SlTPS* genes, accounting for 82% of the family, have available EST or full-length cDNAs (**Table 1**), indicating that these *SlTPS* genes are expressed normally in tomato plants. Phylogenetic tree analysis of the predicted protein sequences with *Arabidopsis* and rice TPSs indicated that the tomato SlTPSs can be classified into two main clades (**Figure 1B**). SlTPS2 and SlTPS8 belong to Clade I but both of them belong to Clade Ia, along with *Arabidopsis* AtTPS1 and rice OsTPS1 (**Figure 1B**). The remaining 8 SlTPSs, including SlTPS1, SlTPS3, SlTPS4, SlTPS5, SlTPS6, SlTPS7, SlTPS9, and SlTPS10, are members of Clade II (**Figure 1B**), which can be further classified into 5 subclades, Clade IIa-e (Yang et al., 2012; Henry et al., 2014).

All of the 8 identified SlTPP proteins contain TPP domain but lack TPS domain (**Table 1**; **Figure 1A**). Four of these *SlTPP* genes including *SlTPP2*, *SlTPP3*, *SlTPP4*, and *SlTPP8*, accounting for 50% of the family, have available ESTs or full-length cDNAs (**Table 1**), indicating that these *SlTPP* genes are expressed in tomato plants. Phylogenetic tree analysis with *Arabidopsis* and rice TPPs revealed that SlTPPs can be classified into four clades (**Figure 1C**). Each of Clade I and Clade II harbors three SlTPPs (SlTPP3, SlTPP4, and SlTPP6 in Clade I and SlTPP2, SlTPP7 and SlTPP8 in Clade II) (**Figure 1C**). However, SlTPPs in Clade I and Clade II are closely clustered with *Arabidopsis* TPPs (**Figure 1C**). SlTPP5 was clustered with rice OsTPP13, forming Clade IV; however, SlTPP1 did not cluster with any of *Arabidopsis* and rice TPPs, becoming the only member in Clade III (**Figure 1C**). Together with the observations in *Arabidopsis* and maize (Vandesteene et al., 2012; Henry et al., 2014), the divergence of the SlTPPs proteins in the phylogenetic tree (**Figure 1C**) may imply that the *SlTPP* genes were evolved through duplication events after the monocot/dicot split.

Like that in *Arabidopsis*, rice and maize (Ge et al., 2008; Henry et al., 2014; Lunn et al., 2014), the tomato genome contains only one trehalase gene, *SlTRE1* (**Table 1**). The SlTRE1 protein shows 53 and 57% of identity to *Arabidopsis* AtTRE1 and rice OsTRE1, respectively. One EST and one full-length cDNA that match to the predicted *SlTRE1* sequence (**Table 1**) were identified in database, indicating *SlTRE1* is also expressed normally in tomato plants.

### Expression Patterns of Selected *SlTPSs*, *SlTPPs*, and *SlTRE1* in Response to Pathogens and Defense Signaling Hormones

**FIGURE 2:**
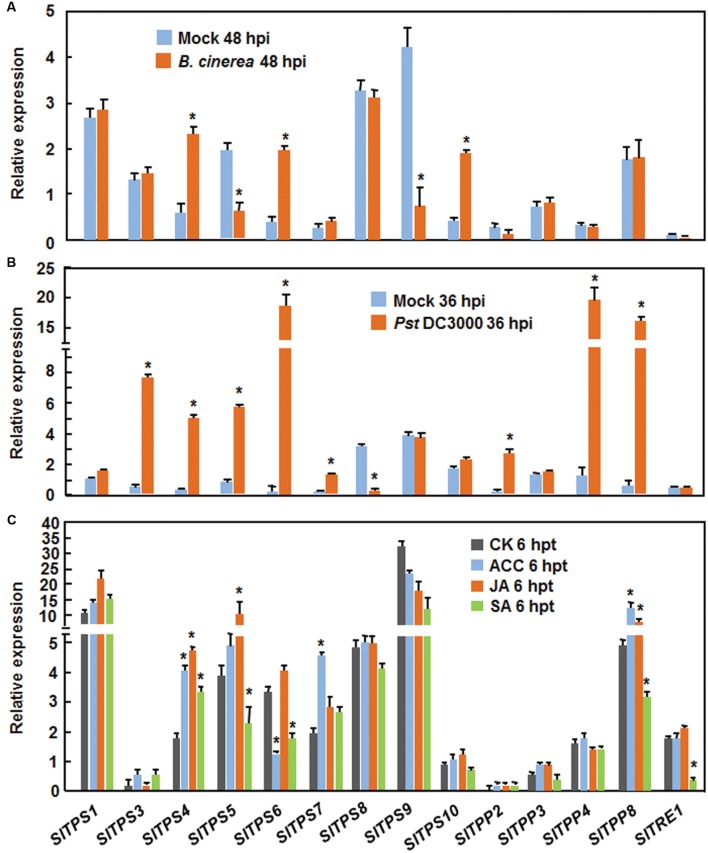
**Expression of selected *SlPTSs*, *SlTPPs*, and *SlTRE1* in responses to infection with *Botrytis cinerea* or *P. syringae* pv. *tomato* DC3000 and to treatments with defense signaling hormones.** **(A)** Expression of selected trehalose-related genes in response to *B. cinerea*. Four-week-old plants were inoculated by foliar spraying with spore suspension of *B. cinerea* or with same volume of buffer as a mock control and leaf samples were collected at 48 h after inoculation for analysis of gene expression. **(B)** Expression of selected trehalose-related genes in response to *Pst* DC3000. Four-week-old plants were inoculated by vacuum infiltration with suspension of *Pst* DC3000 or with 10 mM MgCl_2_ solution as a mock control and leaf samples were collected at 36 h after inoculation for analysis of gene expression. **(C)** Expression of selected trehalose-related genes in response to defense signaling hormones. Tomato plants were treated by foliar spraying of 100 μM SA, 100 μM MeJA, 100 μM ACC or similar volume of solution as a control and leaf samples were collected after 6 h for analysis of gene expression. Expression data were normalized with the value of a reference *SlActin* gene and relative expression was shown as folds of the *SlActin* expression level. Data presented are the means ± SD from three independent experiments and ^∗^ above the columns indicate significant differences at *p* < 0.05 level between the pathogen-inoculated or hormone-treated plants and the mock-inoculated/treated plants.

Nine *SlTPSs* (*SlTPS1*, *SlTPS3*, *SlTPS4*, *SlTPS5*, *SlTPS6*, *SlTPS7*, *SlTPS8*, *SlTPS9*, and *SlTPS10*), 4 *SlTPPs* (*SlTPP2*, *SlTPP3*, *SlTPP4*, and *SlTPP8*) and *SlTRE1*, which have EST or full-length cDNA supports (**Table 1**), were selected for further functional analysis. As a first step, we examined the expression of the selected *SlTPS*, *SlTPS* and *SlTRE* genes in tomato plants at 48 or 36 h after inoculation with *B. cinerea* or *Pst* DC3000, as the pathogens normally colonize and proliferate in the inoculated leaves at these time points (Li et al., 2014b, 2015; Zhang et al., 2014). At 48 h after inoculation with *B. cinerea*, the expression of *SlTPS4*, *SlTPS6*, and *SlTPS10* was significantly upregulated, leading to 3.7∼6.3-fold increases, while the expression of *SlTPS5* and *SlTPS9* was markedly downregulated, resulting in 2.4- and 3.5-fold decrease, respectively, as compared with those in mock-inoculated plants (**Figure 2A**). Expression of other *SlTPSs* (*SlTPS2*, *SlTPS3*, *SlTPS7*, and *SlTPS8*), 4 *SlTPPs* and *SlTRE1* was not affected by *B. cinerea* (**Figure 2A**). At 36 h after inoculation with *Pst* DC3000, the expression of *SlTPS3*, *SlTPS4*, *SlTPS5*, *SlTPS6*, *SlTPS7*, and *SlTPP2*, *SlTPP4*, and *SlTPP8* was significantly upregulated, leading to 5.6∼91.1-fold increases, while the expression of *SlTPS8* was markedly downregulated, resulting in 13.2-folds decrease, respectively, as compared with those in mock-inoculated plants (**Figure 2B**). Expression of *SlTPS1*, *SlTPS9*, *SlTPS10*, *SlTPP3*, and *SlTRE1* was not affected by *Pst* DC3000 (**Figure 2B**). The responsiveness of these selected *SlTPSs*, *SlTPPs*, and *SlTRE1* to defense signaling hormones such as salicylic acid (SA), methyl jasmonate (MeJA), and 1-amino cyclopropane-1-carboxylic acid (ACC, a precursor of ET) was also analyzed. As shown **Figure 2C**, the expression of *SlTPS4*, *SlTPS5*, *SlTPS6*, *SlTPS7*, *SlTPP8*, and *SlTRE1* was affected by at least one of the defense signaling hormones at 6 h after treatment, while the expression of *SlTPS1*, *SlTPS3*, *SlTPS8*, *SlTPS9*, *SlTPS10*, *SlTPP2*, *SlTPP3*, and *SlTPP4* was not affected by any of the defense signaling hormones. Among the genes whose expression was affected by defense signaling hormones, the expression of *SlTPS4* was significantly upregulated by three defense signaling hormones (**Figure 2C**). In particular, SA suppressed the expression of *SlTPS6* and *SlTRE1* while JA induced the expression of *SlTPS5* and *SlTPP8* (**Figure 2C**). ACC induced the expression of *SlTPS7* and *SlTPP8* but suppressed the expression of *SlTPS6* (**Figure 2C**). Taken together, these data indicate that some of the 14 selected *SlTPS*, *SlTPP*, and *SlTRE1* genes responded with different expression patterns to infection of *B. cinerea* or *Pst* DC3000 and to at least one of the defense signaling hormones.

### Silencing of 14 Selected *SlTPSs*, *SlTPPs*, and *SlTRE1* Genes in Tomato

**FIGURE 3:**
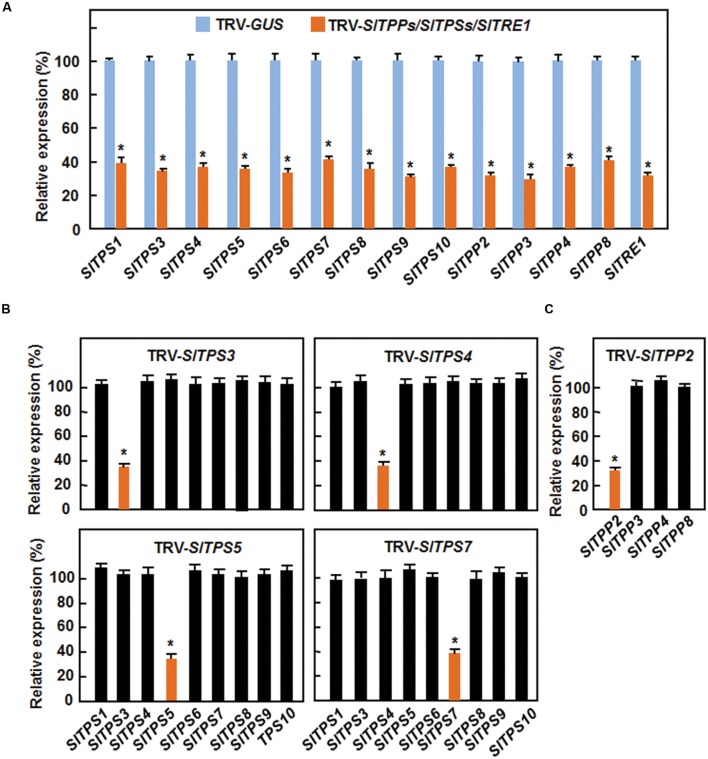
**Silencing efficiency and specificity for selected *SlTPS*, *SlTPP* and *SlTRE1* genes in VIGS-infiltrated plants.** **(A)** Silencing efficiency for each of the selected trehalose-related genes in corresponding VIGS-infiltrated plants. **(B,C)** Silencing specificity for 4 *SlTPS* genes and for *SlTPP2*. Ten-day-old tomato plants were infiltrated with agrobacteria carrying TRV-SlTPSs/SlTPPs/SlTRE1 or TRV-GUS constructs and leaf samples were collected at 4 weeks after agroinfiltration. Transcript levels for the selected trehalose-related genes were analyzed by qRT-PCR using a tomato *SlActin* gene as an internal control. Expression levels of the selected trehalose-related genes in TRV-SlTPSs/SlTPPs/SlTRE1-infiltrated plants were shown as percentages of the levels in TRV-GUS-infiltrated plants. Data presented are the means ± SD from three independent experiments and ^∗^ above the columns indicate significant differences at *p* < 0.05 level between the TRV-SlTPSs/SlTPPs/SlTRE1-infiltrated and TRV-GUS-infiltrated plants.

To explore the possible involvement of the trehalose-related genes in disease resistance, we manipulated the endogenous expression levels of each of the 14 selected *SlTPS*, *SlTPP*, and *SlTRE1* genes by VIGS approach and examined their effects on disease resistance to *B. cinerea* or *Pst* DC3000. To do this, we first examined the silencing efficiency and specificity of the designed VIGS fragments for each of the selected *SlTPS*, *SlTPP*, and *SlTRE1* genes. Standard VIGS protocol was applied to 2-week-old tomato plants (Liu et al., 2002; Li et al., 2014a,b) and the silencing efficiency was analyzed at 4 weeks after VIGS treatment. In our VIGS experiments, plants infiltrated with a TRV-PDS construct as positive controls started to display bleaching symptom on newly developed leaves at 10 days and >90% of the plants showed bleaching symptom at 4 weeks after VIGS infiltration. As shown in **Figure 3A**, the transcript levels for the target genes in corresponding TRV-SlTPSs-, TRV-SlTPPs-, or TRV-SlTRE1-infiltrated plants were 28–39% of those in TRV-GUS-infiltrated plants, indicating that the silencing efficiency for these trehalose-related genes was 61–72%. We also examined the silencing specificity of *SlTPS3*, *SlTPS4*, *SlTPS5*, *SlTPS7*, and *SlTPP2*, whose silencing led to altered resistance to *B. cinerea* or *Pst* DC3000 (see below), by comparing the transcript levels of the target gene and its relative family members in TRV-SlTPS3-, TRV-SlTPS4-, TRV-SlTPS5-, TRV-SlTPS7-, and TRV-SlTPP2-infiltrated plants. Compared with those in the TRV-GUS-infiltrated plants, the transcript levels for *SlTPS3*, *SlTPS4*, *SlTPS5*, *SlTPS7*, and *SlTPP2* were significantly decreased in TRV-SlTPS3-, TRV-SlTPS4-, TRV-SlTPS5-, TRV-SlTPS7-, and TRV-SlTPP2-infiltrated plants, respectively, but the transcript levels of other family members were comparable (**Figures 3B,C**). These data demonstrate that silencing of *SlTPS3*, *SlTPS4*, *SlTPS5*, *SlTPS7*, or *SlTPP2* only downregulated the expression of itself but did not affect the expression of other *SlTPS* or *SlTPP* genes in the same family.

During our studies, we noted that the *SlTPS7*- and *SlTPS8*-silenced plants displayed reduced plant heights, resulting in 25 and 33% of reduction at 4 weeks after VIGS infiltration, as compared with the TRV-GUS-infiltrated plants (**Supplementary Figure S1**). These results indicate that *SlTPS7* and *SlTPS8* may have functions in regulation of vegetative growth in tomato. However, silencing of each of other *SlTPS* (*SlTPS1*, *SlTPS3*, *SlTPS4*, *SlTPS5*, *SlTPS6*, *SlTPS9*, and *SlTPS10*), *SlTPP* (*SlTPP2*, *SlTPP3*, *SlTPP4*, and *SlTPP8*) and *SlTRE1* genes did not affect vegetative growth of the silenced plants (data not shown).

### Silencing of *SlTPS3*, *SlTPS4*, or *SlTPS7* Led to Decreased Resistance to *B. cinerea*

**FIGURE 4:**
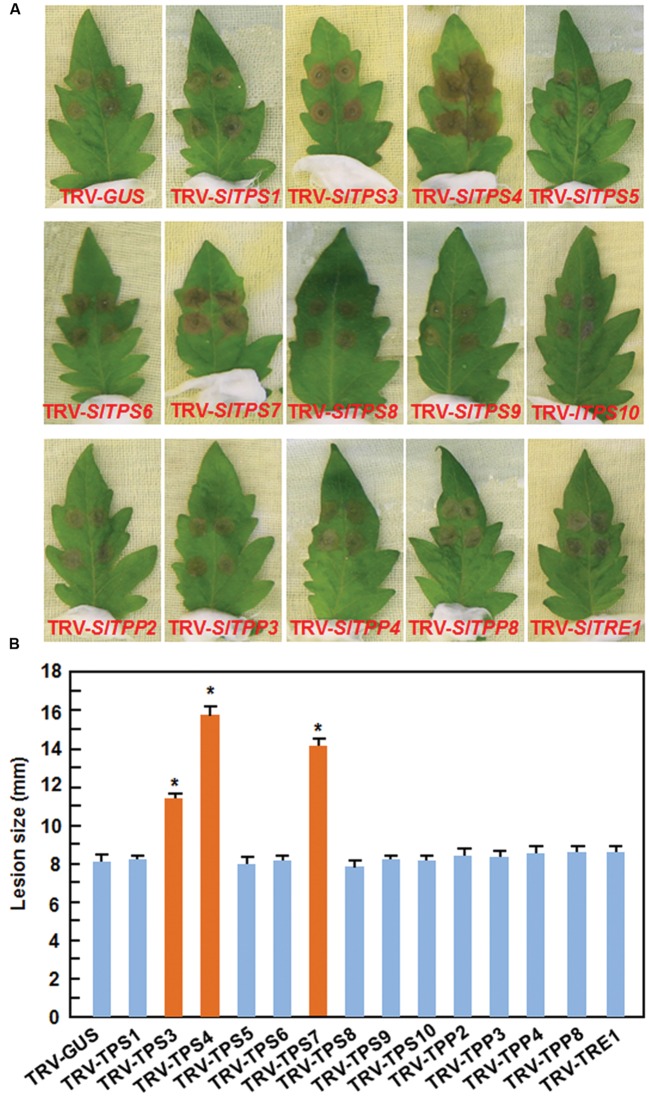
**Silencing of *SlTPS3*, *SlTPS4*, and *SlTPS7* led to decreased resistance against *B. cinerea* in detached leaf assays.** Ten-day-old plants were infiltrated with agrobacteria carrying TRV-SlTPSs/SlTPPs/SlTRE1 or TRV-GUS constructs and leaves were collected at 4 weeks after agroinfiltration for disease assays with *B. cinerea*. **(A)** Disease symptom on representative leaves from the TRV-SlTPSs/SlTPPs/SlTRE1-infiltrated and TRV-GUS-infiltrated plants. **(B)** Size of lesions on leaves from the TRV-SlTPSs/SlTPPs/SlTRE1-infiltrated and TRV-GUS-infiltrated plants. Detached leaf disease assays were performed by dropping 5 μL of spore suspension onto the detached leaves and lesion sizes were measured 3 days after inoculation. At least 10 leaves from 10 individual TRV-SlTPSs/SlTPPs/SlTRE1- and TRV-GUS-infiltrated plants were used in each of three independent experiments. Similar results were obtained in independent experiments **(A)**. Data presented in **(B)** are the means ± SD from three independent experiments and ^∗^ above the columns indicate significant differences at *p* < 0.05 level between the TRV-SlTPSs/SlTPSs/SlTRE1-infiltrated and TRV-GUS-infiltrated plants.

**FIGURE 5:**
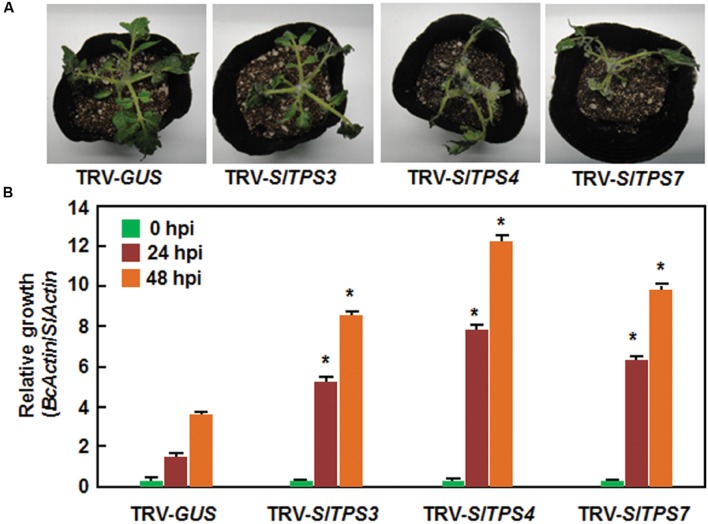
**Silencing of *SlTPS3*, *SlTPS4*, and *SlTPS7* led to decreased resistance against *B. cinerea* in whole plant assays.** **(A)** Disease phenotype of representative TRV-SlTPS3-, TRV-SlTPS4-, TRV-SlTPS7-, and TRV-GUS-infiltrated plants. Photos were taken at 4 days after inoculation. **(B)** *In planta* growth of *B. cinerea* in inoculated TRV-SlTPS3-, TRV-SlTPS4-, TRV-SlTPS7-, and TRV-GUS-infiltrated plants. Whole plant disease assays were done by foliar spraying with spore suspension at 4 weeks after VIGS infiltration. Transcript levels for *B. cinerea BcActinA* and tomato *SlActin* genes in *B. cinerea*-inoculated plants were analyzed using qRT-PCR and *in planta* relative growth of *B. cinerea* was shown as ratios of transcript levels of *BcActinA*/*SlActin*. Similar results were obtained in independent experiments **(A)** and data presented in **(B)** are the means ± SD from three independent experiments. ^∗^ above the columns indicate significant differences at *p* < 0.05 level between the TRV-SlTPS3/4/7-infiltrated and TRV-GUS-infiltrated plants.

To examine the possible involvement of the selected *SlTPS*, *SlTPP*, and *SlTRE1* genes in resistance to *B. cinerea*, a necrotrophic fungal pathogen, we used two different methods, detached leaf disease assays for fast evaluation and whole plant disease assays for confirmation, to compare the disease phenotype and *in planta* fungal growth in the TRV-SlTPS/SlTPP/SlTRE1-infiltrated plants with those in the TRV-GUS-infiltrated plants. In the detached leaf disease assays, typical small necrotic lesions were seen at 2 days post inoculation (dpi). At 3 dpi, sizes of the lesions on leaves from TRV-SlTPS1-, TRV- SlTPS5-, TRV-SlTPS6-, TRV-SlTPS8-, TRV-SlTPS9-, TRV-SlT PS10-, TRV-SlTPP2-, TRV-SlTPP3-, TRV-SlTPP4-, TRV-SlT PP8-, and TRV-SlTRE1-infiltrated plants were similar to that in the TRV-GUS-infiltrated plants (**Figures 4A,B**), indicating that *SlTPS1*, *SlTPS5*, *SlTPS6*, *SlTPS8*, *SlTPS9*, *SlTPS10*, *SlTPP2*, *SlTPP3*, *SlTPP4*, *SlTPP8*, and *SlTRE1* may not be involved in resistance to *B. cinerea*. By contrast, sizes of the lesions on leaves from the TRV-SlTPS3-, TRV-SlTPS4-, and TRV-SlTPS7-infiltrated plants were significantly increased (**Figure 4A**), leading to 38, 97, and 75% of increases, respectively, than those in the TRV-GUS-infiltrated plants at 3 dpi (**Figure 4B**). To confirm this observation, we further evaluated the disease phenotype and measured *in planta* fungal growth of *B. cinerea* in the TRV-SlTPS3-, TRV-SlTPS4-, and TRV-SlTPS7-infiltrated plants using whole plant disease assays. As shown in **Figure 5A**, the TRV-SlTPS3-, TRV-SlTPS4-, and TRV-SlTPS7-infiltrated plants had larger necrotic areas and leaf maceration at 5 dpi, as compared with the TRV-GUS-infiltrated plants. Accordingly, *in planta* growth of *B. cinerea*, as represented by the transcript levels of the *B. cinerea BcActinA* gene, in leaf tissues of the TRV-SlTPS3-, TRV-SlTPS4-, and TRV-SlTPS7-infiltrated plants was significantly increased, showing three–four times higher than that in the TRV-GUS-infiltrated control plants at 24 and 48 hpi (**Figure 5B**). Taken together, these results demonstrate that silencing of *SlTPS3*, *SlTPS4*, or *SlTPS7* deteriorated the resistance of tomato plants against *B. cinerea* and supported more growth of *B. cinerea* in the TRV-SlTPS3-, TRV-SlTPS4-, and TRV-SlTPS7-infiltrated plants.

**FIGURE 6:**
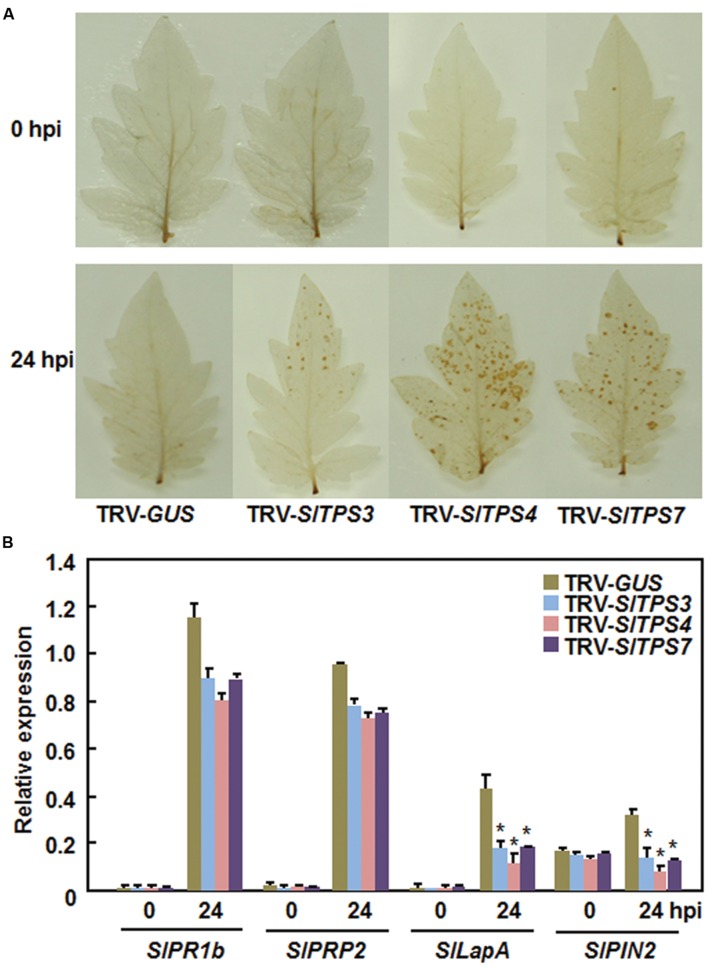
**Silencing of *SlTPS3*, *SlTPS4*, and *SlTPS7* increased accumulation of H_2_O_2_ and decreased the expression levels of JA/ET signaling-responsive defense-related genes after infection with *B. cinerea*.** Whole plant disease assays were done by foliar spraying with spore suspension at 4 weeks after VIGS infiltration and leaf samples were collected at 24 h after inoculation. **(A)** Accumulation of H_2_O_2_, as detected by DAB staining, in TRV- SlTPS3-, TRV-SlTPS4-, TRV-SlTPS7-, and TRV-GUS-infiltrated plants after infection of *B. cinerea*. **(B)** Expression patterns of selected defense-related genes in TRV-SlTPS3-, TRV-SlTPS4-, TRV-SlTPS7-, and TRV-GUS-infiltrated plants after infection of *B. cinerea*. Expression data for the selected defense-related genes in TRV-SlTPS3-, TRV-SlTPS4-, TRV-SlTPS7-, and TRV-GUS-infiltrated plants were normalized with the value of a reference *SlActin* gene and relative expression was shown as folds of the *SlActin* expression level. Similar results were obtained in independent experiments **(A)** and data presented in **(B**) are the means ± SD from three independent experiments. ^∗^ above the columns indicate significant differences at *p* < 0.05 level between the TRV-SlTPS3/4/7-infiltrated and TRV-GUS-infiltrated plants.

To explore the possible mechanism by which silencing of *SlTPS3*, *SlTPS4*, or *SlTPS7* led to decreased resistance against *B. cinerea*, we analyzed and compared the defense responses including accumulation of reactive oxygen species (ROS) and expression of defense-related genes in the TRV-SlTPS3-, TRV-SlTPS4-, and TRV-SlTPS7-infiltrated plants before and after infection of *B. cinerea*. At 0 h, no accumulation of H_2_O_2_ was observed in the leaves from the TRV-SlTPS3-, TRV-SlTPS4-, or TRV-SlTPS7-infiltrated plants and the TRV-GUS-infiltrated plants (**Figure 6A**). However, significant accumulation of H_2_O_2_ was observed in leaves of the TRV-SlTPS3-, TRV-SlTPS4-, and TRV-SlTPS7-infiltrated plants, while only slight accumulation of H_2_O_2_ was detected in leaves of TRV-GUS-infiltrated plants, at 24 h after inoculation with *B. cinerea* (**Figure 6A**). Similarly, the expression of some selected SA signaling-responsive defense-related genes *SlPR1b* and *SlPRP2* and JA/ET signaling-responsive defense-related genes *SlLapA* and *SlPIN2* was comparable between the TRV-SlTPS3-, TRV-SlTPS4-, or TRV-SlTPS7-infiltrated plants and the TRV-GUS-infiltrated plants before infection of *B. cinerea* (**Figure 6B**). Although the expression of these SA signaling-responsive and JA/ET signaling-responsive defense-related genes was upregulated significantly by infection of *B. cinerea*; however, the expression levels of *SlPR1b* and *SlPRP2* were slightly reduced while the expression levels of *SlLapA* and *SlPIN2* were significantly decreased in TRV-SlTPS3-, TRV-SlTPS4-, and TRV-SlTPS7-infiltrated plants, as compared with those in the TRV-GUS-infiltrated plants, at 24 h (**Figure 6B**). Together, these data indicate that silencing of *SlTPS3*, *SlTPS4*, or *SlTPS7* deregulated ROS accumulation and attenuated the expression of the JA/ET signaling-responsive defense-related genes upon infection of *B. cinerea*.

### Silencing of *SlTPS4* Decreased but Silencing of *SlTPS5* or *SlTPP2* Increased the Resistance against *Pst* DC3000

**FIGURE 7:**
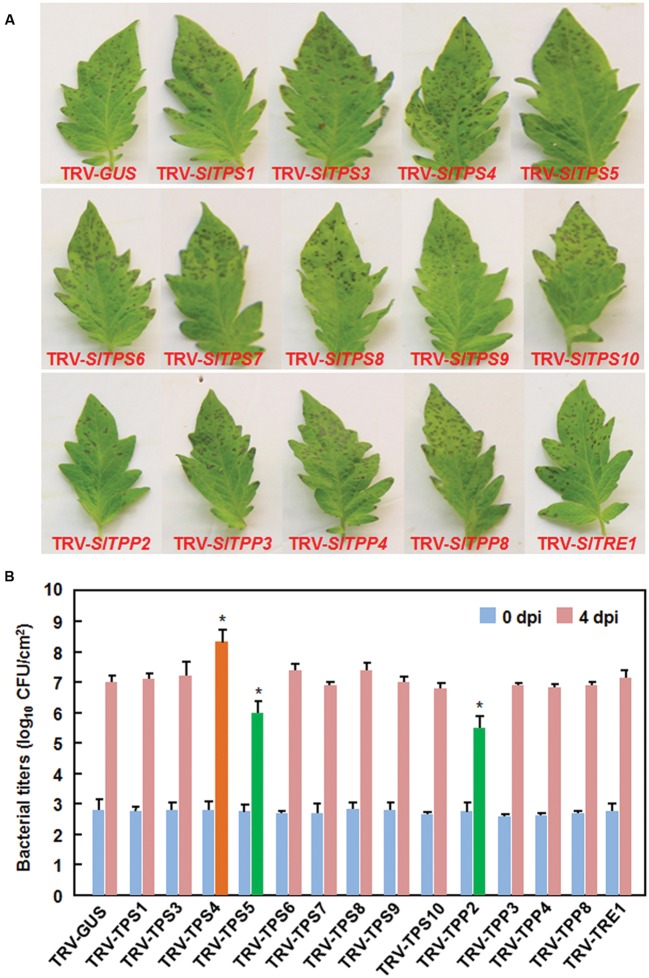
**Silencing of *SlTPS4* decreased and silencing of *SlTPS5* or *SlTPP2* increased the resistance against *P. syringae* pv. *tomato* DC3000.** Ten-day-old plants were infiltrated with agrobacteria carrying TRV-SlTPSs/SlTPPs/SlTRE1 or TRV-GUS constructs and disease assays were carried out at 4 weeks after agroinfiltration. The TRV-SlTPSs/SlTPPs/SlTRE1- and TRV-GUS-infiltrated plants were inoculated by vacuum infiltration with suspension of *P. syringae* pv. *tomato* DC3000. **(A)** Disease symptom on representative leaves from TRV-SlTPSs/SlTPPs/SlTRE1- and TRV-GUS-infiltrated plants at 4 days after inoculation with *P. syringae* pv. *tomato* DC3000. **(B)** Bacterial population in inoculated leaves of the TRV-SlTPSs/SlTPPs/SlTRE1- and TRV-GUS-infiltrated plants. Leaf samples were collected at 0 and 4 days after inoculation and bacterial population was measured. Similar results were obtained in independent experiments **(A)**. Data presented in **(B)** are the means ± SD from three independent experiments and ^∗^ above the columns indicate significant differences at *p* < 0.05 level between the TRV-SlTPSs/SlTPSs/SlTRE1-infiltrated and TRV-GUS-infiltrated plants.

We next examined the possible involvement of the selected *SlTPS*, *SlTPP*, and *SlTRE1* genes in resistance against *Pst* DC3000, a (hemi)biotrophic bacterial pathogen, by comparing the disease phenotype and *in planta* bacterial growth in the TRV-target SlTPS/SlTPP/SlTRE1-infiltrated plants with those in the TRV-GUS-infiltrated plants. At 3 dpi, the TRV-SlTPS4-infiltrated plants displayed more severe disease while the TRV-SlTPS5- and TRV-SlTPP2-infiltrated plants showed less severe disease, as compared with the TRV-GUS-infiltrated plants (**Figure 7A**). At 4 dpi, the bacterial population (2.24 × 10^8^ colony-forming unit (cfu)/cm^2^ leaf tissues) in leaves of TRV-SlTPS4-infiltrated was 23.46 times higher than that in the TRV-GUS-infiltrated plants (9.55 × 10^6^ cfu/cm^2^ leaf tissues). By contrast, the bacterial populations in leaves of the TRV-SlTPS5- and TRV-SlTPP2-infiltrated plants (1.07 × 10^6^ cfu/cm^2^ leaf tissues and 3.63 × 10^5^ cfu/cm^2^ leaf tissues, respectively) were 7.93 and 25.31 times less than that in the TRV-GUS-infiltrated plants, respectively, at 4 dpi (**Figure 7B**). Disease symptom on and bacterial growth in leaves from TRV-SlTPS1-, TRV-SlTPS3-, TRV-SlTPS6-, TRV-SlTPS7-, TRV-SlTPS8-, TRV-SlTPS9-, TRV-SlTPS10-, TRV-SlTPP3-, TRV-SlTPP4-, TRV-SlTPP8-, and TRV-SlTRE1-infiltrated plants were similar to those in the TRV-GUS-infiltrated plants (**Figures 7A,B**), indicating that *SlTPS1*, *SlTPS3*, *SlTPS6*, *SlTPS7*, *SlTPS8*, *SlTPS9*, *SlTPS10*, *SlTPP3*, *SlTPP4*, *SlTPP8*, and *SlTRE1* may not be involved in resistance against *Pst* DC3000. These results indicate that silencing of *SlTPS4* decreased the resistance while silencing of *SlTPS5* or *SlTPP2* increased the resistance against *Pst* DC3000 in tomato.

**FIGURE 8:**
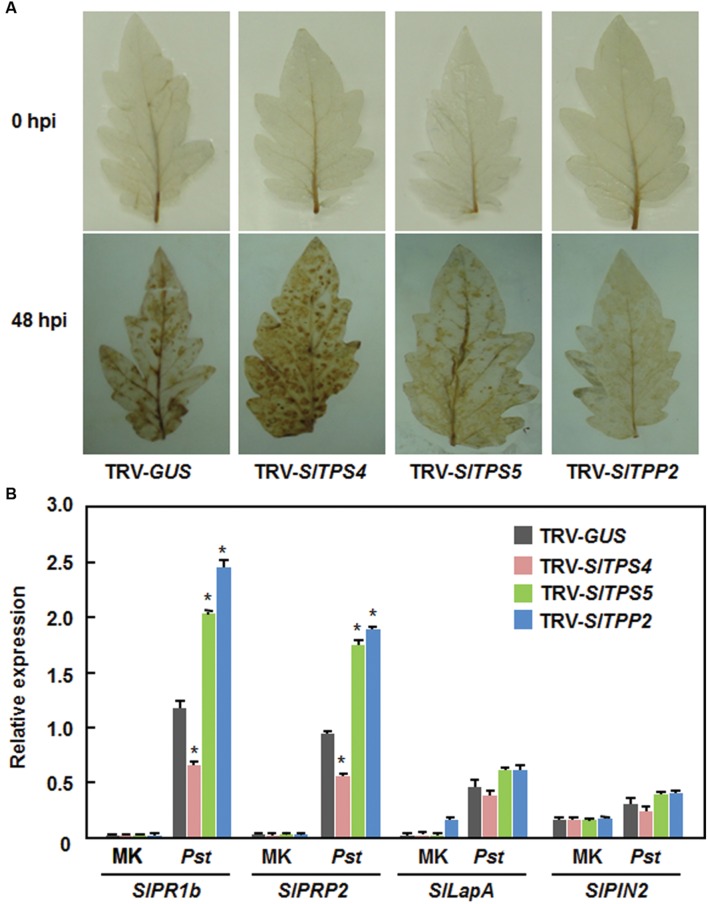
**Silencing of *SlTPS4*, *SlTPS5*, and *SlTPP2* affected H_2_O_2_ accumulation and expression of SA signaling-responsive defense-related genes after infection with *P. syringae* pv. *tomato* DC3000.** Whole plant disease assays were done by vacuum infiltration with suspension of *P. syringae* pv. *tomato* DC3000 at 4 weeks after VIGS infiltration and leaf samples were collected at 24 h after inoculation. **(A)** Accumulation of H_2_O_2_, as detected by DAB staining, in TRV-SlTPS4-, TRV-SlTPS5-, TRV-SlTPP2-, and TRV-GUS-infiltrated plants after infection of *Pst* D3000. **(B)** Expression patterns of selected defense-related genes in TRV-SlTPS4-, TRV-SlTPS5-, TRV-SlTPP2-, and TRV-GUS-infiltrated plants after infection of *Pst* DC3000. Expression data for the selected defense-related genes in TRV-SlTPS4-, TRV-SlTPS5-, TRV-SlTPP2-, and TRV-GUS-infiltrated plants were normalized with the value of a reference *SlActin* gene and relative expression levels were shown as folds of the *SlActin* expression level. Similar results were obtained in independent experiments **(A)** and data presented in **(B)** are the means ± SD from three independent experiments. ^∗^ above the columns indicate significant differences at *p* < 0.05 level between the TRV-SlTPS4/SlTPS5/SlTPP2-infiltrated and TRV-GUS-infiltrated plants.

We also analyzed and compared the accumulation of ROS and expression of defense-related genes in the TRV-SlTPS4-, TRV-SlTPS5-, and TRV-SlTPP2-infiltrated plants before and after infection of *Pst* DC3000 to gain insights into the possible mechanism that silencing of *SlTPS4*, *SlTPS5*, or *SlTPP2* affected the resistance against *Pst* DC3000. Before infection of *Pst* DC3000, no significant accumulation of H_2_O_2_ was seen in leaves of the TRV-SlTPS4-, TRV-SlTPS5-, TRV-SlTPP2-, and TRV-GUS-infiltrated plants (**Figure 8A**). However, at 3 dpi, significant accumulation of H_2_O_2_ was observed in leaves of the TRV-SlTPS4-infiltrated plants, while less accumulation of H_2_O_2_ in leaves of the TRV-SlTPS5- and TRV-TPP2-infiltrated plants was detected, as compared with that in leaves of the TRV-GUS-infiltrated plants (**Figure 8A**). Similarly, the expression of defense-related genes *SlPR1b*, *SlPRP2*, *SlLapA*, and *SlPIN2* was comparable between the TRV-SlTPS4-, TRV-SlTPS5-, or TRV-SlTPP2-infiltrated plants and the TRV-GUS-infiltrated plants before infection of *Pst* DC3000 (**Figure 8B**). The expression levels of *SlPR1b* and *SlPRP2* in the TRV-SlTPS4-infiltrated plants were decreased while the expression levels of these two defense-related genes in the TRV-SlTPS5- and TRV-SlTPP2-infiltrated plants were significantly increased, as compared with those in the TRV-GUS-infiltrated plants, at 2 dpi after inoculation with *Pst* DC3000 (**Figure 8B**). However, the expression levels of *SlLapA* and *SlPIN*2 in the TRV-SlTPS4-, TRV-SlTPS5-, or TRV-SlTPP2-infiltrated plants were not significantly affected, as compared with those in the TRV-GUS-infitlrated plants, at 2 dpi after inoculation with *Pst* DC3000 (**Figure 8B**). These data indicate that silencing of *SlTPS4* attenuated while silencing of *SlTPS5* or *SlTPP2* strengthened the expression of the SA signaling-responsive defense-related genes upon infection of *Pst* DC3000.

### Silencing of *SlTPS3*, *SlTPS4*, *SlTPS5*, *SlTPS7*, or *SlTPP2* Affected Trehalose Content in Tomato Plants with or without Pathogen Infection

**FIGURE 9:**
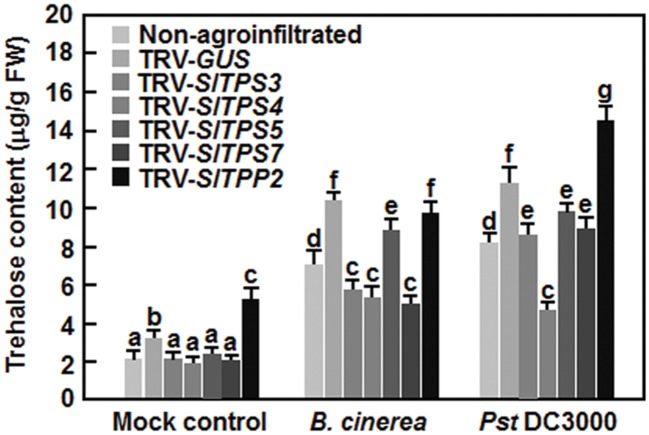
**Changes of trehalose contents in *SlTPS3*-, *SlTPS4*-, *SlTPS5*-, *SlTPS7*-, and *SlTPP2*-silenced plants with or without pathogen infection.** Ten-day-old plants were infiltrated with agrobacteria carrying TRV-SlTPS3, TRV-SlTPS4, TRV-SlTPS5, TRV-SlTPS7, TRV-SlTPP2, or TRV-GUS construct and were inoculated with spore suspension of *B. cinerea* or bacterial suspension of *Pst* DC3000 at 4 weeks after VIGS infiltration. Leaf samples were collected for measurement of trehalose contents at 48 h after pathogen inoculation. Data presented are the means ± SD from three independent experiments and different letters above the columns indicate significant differences at *p* < 0.05 level.

To examine the possible involvement of trehalose in defense response, we analyzed the trehaolse contents in TRV-SlTPS3-, TRV-SlTPS4-, TRV-SlTPS5-, TRV-SlTPS7-, and TRV-SlTPP2-infiltrated plants with or without infection of *B. cinerea* or *Pst* DC3000. At 4 weeks after VIGS infiltration, trehalose content in TRV-GUS-infiltrated plants was 71% higher than that in non-agroinfiltrated plants (**Figure 9**), indicating that infiltrated agrobacteria and/or TRV affected trehalose content in tomato plants. Without pathogen infection, trehalose contents in TRV-SlTPS3-, TRV-SlTPS4-, TRV-SlTPS5-, and TRV-SlTPS7-silenced plants were decreased by 35∼45% while the trehalose content in TRV-SlTPP2-infiltrated plants was increased by 47%, as compared to that in TRV-GUS-silenced plants (**Figure 9**). At 48 h after inoculation, infection of *B. cinerea* or *Pst* DC3000 increased the trehalose contents in non-agroinfiltrated and TRV-GUS-infiltrated plants, leading to 1.9–2.5 and 2.2–3.1-folds of increases by *B. cinerea* and *Pst* DC3000, respectively (**Figure 9**). As compared with those in TRV-GUS-infiltrated plants, trehalose contents in TRV-SlTPS3-, TRV-SlTPS4-, and TRV-SlTPS7-infiltrated plants after infection of *B. cinerea* were decreased by 44–54% while trehalose content in TRV-SlTPS4-infiltrated plants after infection of *Pst* DC3000 was reduced by 58% (**Figure 9**). Trehalose content in TRV-SlTPP2-infiltrated plants was similar to that in TRV-GUS-infiltrated plants after infection of *B. cinerea*, whereas the content was increased by 27% after infection of *Pst* DC3000 (**Figure 9**). These data suggest that silencing of *SlTPS3*, *SlTPS4*, *SlTPS5*, *SlTPS7*, or *SlTPP2* affected trehalose content in tomato plants with or without pathogen infection.

## Discussion

In the present study, we identified 11 *SlTPS*, 8 *SlTPP*, and 1 *SlTRE1* genes in tomato (**Table 1**). The numbers of *SlTPS*, *SlTPP*, and *SlTRE1* genes are similar to those observed in *Arabidopsis* (Leyman et al., 2001; Vandesteene et al., 2012), rice (Ge et al., 2008; Zhang et al., 2011), wheat (Xie et al., 2015), and maize (Henry et al., 2014; Zhou et al., 2014). This indicates that the *SlTPS* and *SlTPP* gene families in tomato are conserved with the *TPS* and *TPP* families in other plants, probably due to the evolution feature that at least the eudicot and many monocot *TPP* genes originate from whole-genome duplications (Vandesteene et al., 2012). Although the biological functions of TPSs, TPPs, and TRE1 in plant growth/development and abiotic stress response have been implicated, direct evidence supporting the roles of TPSs, TPPs, and TRE1 in plant disease resistance is lacking yet. Our VIGS-based functional analyses of 9 *SlTPSs* (82% of the family), 4 *SlTPPs* (50% of the family), and *SlTRE1* revealed that silencing of *SlTPS3*, *SlTPS4*, *SlTPS5*, *SlTPS7*, or *SlTPP2* affected the resistance against *B. cinerea* and *Pst* DC3000, two different pathogens with distinct infection styles. To our knowledge, these findings provide the first lines of evidence supporting the involvement of the trehalose-related genes in plant disease resistance.

In our VIGS assays, the silencing efficiency for individual target gene of the 14 selected *SlTPSs*, *SlTPPs* and *SlTRE1* was estimated to be 61–72% (**Figure 3A**), which is similar to those observed in our previous studies (Li et al., 2014a,b; Liu et al., 2014; Zhang et al., 2014). Silencing specificity of *SlTPS3*, *SlTPS4*, *SlTPS5*, *SlTPS7*, or *SlTPP2* (**Figure 3B**) demonstrates that the altered phenotypes in growth and disease resistance observed in the present study were the consequences of the silencing of specific individual *SlTPS* or *SlTPP* genes. Notably, we observed that silencing of either *SlTPS7* or *SlTPS8* led to inhibition of vegetative growth (**Supplementary Figure S1**), indicating that both SlTPS7 and SlTPS8 have functions in regulation of vegetative growth in tomato. SlTPS8 is phylogenetically closely related to *Arabidopsis* AtTPS1 (**Figure 1B**), which was shown to be essential for vegetative growth (van Dijken et al., 2004; Gómez et al., 2010). Therefore, it is likely that SlTPS8 and AtTPS1 have evolutionary conserved functions in vegetative growth of the *Arabidopsis* and tomato plants. In addition, AtTPS1 was also found to be essential for embryogenesis and flowering (Eastmond et al., 2002; van Dijken et al., 2004; Gómez et al., 2006, 2010; Wahl et al., 2013). The involvement of SlTPS8 in embryogenesis, flowering and other biological processes needs to be further investigated.

It was previously reported that expression of *AtTPS11* was transiently induced by feeding of green peach aphids (Singh et al., 2011). However, some of the trehalose metabolic genes such as rice *OsTPS1* responded with high level of expression by abiotic stress over a period of 3 days after treatment (Ge et al., 2008). Previous studies have shown that the expression of some of *SlTPSs* and *SlTPPs* can be induced by pathogen infection (Brodmann et al., 2002; Golem and Culver, 2003). Diverse spatiotemporal expression patterns were also observed for the 10 *Arabidopsis AtTPP* genes by analyzing promoter GUS/GFP lines (Vandesteene et al., 2012). The differential responsiveness of the selected *SlTPS* and *SlTPP* genes to infection of *B. cinerea* and *Pst* DC3000 and to defense signaling hormones indicates possible functional divergence among the *SlTPSs* and *SlTPPs* in disease resistance against *B. cinerea* and *Pst* DC3000. Moreover, we also noted that some of the *SlTPS* and *SlTPP* genes, which exhibited altered expression in pathogen-infected plants, did not affect the disease resistance to *B. cinerea* or *Pst* DC3000. This can be explained by a common phenomenon that induction of gene expression does not always correlate with an absolute requirement in defense response.

Previous studies have shown that pathogen-induced expression of trehalose-related genes can lead to trehalose accumulation (Brodmann et al., 2002; Hofmann et al., 2010; Piazza et al., 2015) and that transgenic expression of the trehalose metabolic genes can elevate the endogenous trehalose content (Jang et al., 2003; Ge et al., 2008; Singh et al., 2011; Wang et al., 2016). Similarly, we observed that infection of *B. cinerea* or *Pst* DC3000 as well as infiltration with agrobacteria harboring TRV construct induced the trehalose accumulation in tomato plants (**Figure 9**). Most of the *Arabidopsis* Class II TPSs are not active enzymes as revealed by yeast complementation assays (Ramon et al., 2009); however, overexpression of *AtTPS11* and its cotton homologous gene *GhTPS11* in transgenic *Arabidopsis* plants resulted in increased trehalose contents (Singh et al., 2011; Wang et al., 2016), implying that some of the Class II TPSs are active enzymes *in planta* that can catalyze trehalose metabolism. Silencing of *SlTPS3*, *SlTPS4*, *SlTPS5*, or *SlTPS7*, encoding for Class II TPSs, led to decreased trehalose content (**Figure 9**), indicating that SlTPS3, SlTPS4, SlTPS5, and SlTPS7 may be active trehalose metabolic enzymes in tomato. We noted that reduced pathogen-induced trehalose accumulation correlates with the decreased resistance in *SlTPS3*/*4*/*7*-silenced plants to *B. cinerea* and in *SlTPS4*-silenced plants to *Pst* DC3000 while increased pathogen-induced trehalose accumulation associates with enhanced resistance to *Pst* DC3000 in *SlTPP2*-silenced plants (**Figures 4**, **7**, and **9**). This is similar to the observation that *Arabidopsis tps11* mutant plants displayed reduced resistance to aphids while the *AtTPS11*-overexpressing plants contain elevated trehalose content and exhibited increased resistance to aphids (Singh et al., 2011). Notably, silencing of *SlTPS4* or *SlTPS5* had opposite effect on resistance to *Pst* DC3000 (**Figure 7**). Possible explanations include that SlTPS4 and SlTPS5 have differential effects on the *Pst* DC3000-induced trehalose accumulation in *SlTPS4*/*5*-silenced plants (**Figure 9**), or other members of the Class II TPSs may complement the function of SlTPS5 in *SlTPS5*-silenced plants upon infection of *Pst* DC300 via a yet-unknown mechanism. It was reported that *Arabidopsis* AtTPPa and AtTPPg have redundant roles in leaf growth, root hair specification and energy-responses (Van Houtte et al., 2013a). Alternatively, it cannot be ruled out the possibility that altered T6P level due to the silencing of *SlTPS5* in catalyzing the formation of T6P is responsible for resistance to *Pst* DC3000 in *SlTPS5*-silenced plants. Silencing of *SlTPP2* led to increased trehalose content and enhanced resistance to *Pst* DC3000 (**Figures 7** and **9**). This is similar to the observation that mutations in some *Arabidopsis* TPP genes resulted in increased levels of T6P and trehalose (Vandesteene et al., 2012). In addition, the SlTPSs with functions in resistance contribute differentially to resistance against different pathogens. For example, SlTPS4 is required for resistance against both of *B. cinerea* and *Pst DC3000* while SlTPS3 and SlTPS7 have functions in resistance against *B. cinerea* but not to *Pst* DC3000. Collectively, our data demonstrate an important role for trehalose and its metablic genes in resistance against different pathogens.

It was previously shown that trehalose is capable of protecting against damage from ROS such as hydroxyl radicals (Couee et al., 2006; Luo et al., 2008) and that overexpression of yeast *TPS1* in tomato plants increased tolerance to oxidative stress (Cortina and Culianez-Macia, 2005). We observed that the *SlTPS3*/*4*/*7*-silenced plants accumulated excessive level of H_2_O_2_ after infection by *B. cinerea* or *Pst* DC3000 (**Figures 6** and **8**). ROS accumulated during the late stage may favor for the development of diseases caused by necrotrophic pathogens such as *B. cinerea* and (hemi)biotrophic pathogens like *Pst* DC3000 (Govrin and Levine, 2000; Govrin et al., 2006; Temme and Tudzynski, 2009; Ishiga et al., 2012; Mengiste, 2012). Thus, it is likely that deregulation of ROS accumulation caused by silencing of *SlTPS3*, *SlTPS4*, and *SlTPS7* may be responsible for the decreased resistance against *B. cinerea* and *Pst* DC3000 in *SlTPS3*/*4*/*7*-silenced plants. On the other hand, the expression of SA signaling-responsive defense-related genes such as *SlRP1b* and *SlRPP2* and JA/ET signaling-responsive defense-related genes *SlLapA* and *SlPIN2* was attenuated in the *SlTPS3*-, *SlTPS4*-, and *SlTPS7*-silenced plants after infection of *Pst* DC3000 or *B. cinerea*, respectively (**Figures 6** and **8**). This may also be due to the reduced level of trehalose in the *SlTPS3*-, *SlTPS4*-, and *SlTPS7*-silenced plants (**Figure 9**), because exogenous trehalose was found to induce the expression of defense-related genes in wheat and citrus (Tayeh et al., 2014; Piazza et al., 2015). It is therefore likely that the reduced trehalose content may be responsible for deregulation of ROS accumulation and attenuated expression of defense-related genes in the *SlTPS3*-, *SlTPS4*-, and *SlTPS7*-silenced plants. However, this hypothesis cannot be used to explain the mechanism for the increased resistance against *Pst* DC3000 in the *SlTPP2*-silenced plants, which had elevated trehalose content but had decreased accumulation of H_2_O_2_ and upregulated expression of the defense-related genes after infection with *Pst* DC3000 (**Figure 8**). The facts that SA and JA affected the expression of *SlTPS3*, *SlTPS4*, *SlTPS5*, *SlTPS7*, and *SlTPP2* (**Figure 2C**) and that silencing of these genes also affected the expression of pathogen-induced defense genes (**Figures 6** and **8**) may indicate that trehalose or its metabolism act downstream of the SA and JA. This can be verified further by testing whether SA or JA can rescue the reduced resistance phenotype in the *SlTPS3/4/5/7*- and *SlTPP2*-silenced plants.

It was previously reported that *P. brassica*-induced expression of *AtTRE1* acts as a defense response to limit trehalose accumulation (Brodmann et al., 2002; Gravot et al., 2011) and overexpression of *AtTRE1* improves drought stress tolerance in *Arabidopsis* (Van Houtte et al., 2013b). By contrast, the expression of *SlTRE1* was not induced by both of *B. cinerea* and *Pst* DC3000 (**Figure 2**) and silencing of *SlTRE1* did not affect the resistance against *B. cinerea* and *Pst* DC3000 (**Figures 4**, **5** and **7**), indicating that SlTRE1 may not be involved in disease resistance against these two pathogens. Interestingly, *B. cinerea* Δtre1 mutant showed elevated trehalose content but showed similar pathogenicity to wild-type strain (Doehlemann et al., 2006). Thus, it is likely that TRE1 has limited function in tomato-*B. cinerea* interaction, although trehalose serves as a stress protectant and as a significant but not essential carbon source for conidial germination in *B. cinerea* (Doehlemann et al., 2006).

## Author Contributions

Conceived and designed the experiments: FS and HZ. Performed the experiments: HZ, DL, LH, YH, SL, LT, YD, ZC, and LH. Analyzed the data: FS and HZ. Contributed to reagents/materials/analysis tools: HZ and DL. Wrote the paper: FS and HZ.

## Conflict of Interest Statement

The authors declare that the research was conducted in the absence of any commercial or financial relationships that could be construed as a potential conflict of interest.

## Footnotes

1 http://solgenomics.net

2 http://www.ncbi.nlm.nih.gov/Structure/cdd/wrpsb.cgi

3 http://myhits.isb-sib.ch/cgi-bin/motif_scan
