# Supplementary material for: Virus-Induced Gene Silencing-Based Functional Analyses Revealed the Involvement of Several Putative Trehalose-6-Phosphate Synthase/Phosphatase Genes in Disease Resistance against Botrytis cinerea and Pseudomonas syringae pv. tomato DC3000 in Tomato
Source: Front Plant Sci. 2016 Aug 4;7:1176. doi: 10.3389/fpls.2016.01176 (PMC4972837; doi:10.3389/fpls.2016.01176)
Supplement: Supplementary file 1 [file Data_Sheet_1.DOC]

***Supplementary Materials***

**Virus-induced Gene Silencing-based Functional Analyses Revealed the Involvement of Several Putative Trehalose-6-Phosphate Synthase/Phosphatase Genes in Disease Resistance against *Botrytis cinerea* and *Pseudomonas syringae* pv. *tomato* DC3000 in Tomato**

***Huijuan Zhang^1, 2^, Yongbo Hong^2^, Lei Huang^2^, Shixia Liu^2^, Limei Tian^2^, Yi Dai^2^, Zhongye Cao^2^, Lihong Huang^2^, Dayong Li^2^,Fengming Song^2^****

*^1^ College of Life Science, Taizhou University, Taizhou, Zhejiang 318001, P. R. China; ^2^National Key Laboratory for Rice Biology, Institute of Biotechnology, Zhejiang University, Hangzhou 310058, P. R. China*

***Correspondence:**

Dr. Fengming Song

Email: fmsong@zju.edu.cn

**Supplementary file 1. VIGS sequences for *SlTPSs*, *SlTPPs* and *SlTRE1***

Note: 5’-UTR sequences are in lowercase.

***SlTPS1* (Solyc01g005210)**

atgttaggagcttactatagagagtgtgtgttttggtttggtggatttggtgttgttagttagtgaaaagaatttttttataaaaaaagATGGTGTCAAGATCCTATTCCAATCTCTTGGAGCTGGCCTCAGGTGAGGCTCCTTCGCCGTCTTTCGGGCGAATGAGCCGGAGGATTCCACGTGTTATGACCGTCGCTGGGATAATGTCTGATCTGGATGATGATGGATCGGAGAGCGTTTGCTCCGATCCATCATCATCTTCAGCTCAAAAAGATAGGATAATTGTTGTAGCTAATCAGCTGCCAATTAGAGTACAAAGAAAAACTGATGGCAGTAAAGGATGGTTATTCAGCTGGGATGAGAATTCA

***SlTPS3* (Solyc02g072150)**

gtgatgtatgtggattgagttttttgaagagaaaaaaagtttctgtttggtctgttttctaatttgaggatctgctgaaggagtttttgcaaataaagggcttgtaaagATGATGTCCAAATCTTATACCAACTTGCTAGATCTAGCATCTGGGAATTTCCCGGTGATGGGGCGGGAAAAGAAGCGGCTGCCACGGGTAATGACAGTTGCTGGAGTTATTTCTGAGCTTGATGATGATCAAGCTAATAGTGTTACATCAGATGTTCCATCATCAATTATTGTAGATCGAATAATTATAGTGGCTAATCAGCTCCCTGTAAAAGCTAAGCGTAGGTCGGATAATAAAGGATGGAATTTTAGTTGGGATGAGGATTCGTT

***SlTPS4* (Solyc04g025940)**

acacactttcctttggtttcacatatctcagcagaacttgtatgatataacgtaagctaagttctacttcagagacATGGCATCAAGATCTAGTGCAAACTTTTTCGACTTGGCATCTGAGGACATACTGGATATACATCAGACTCCTAGAGCACTTCCGCGTATGATGACTGTTCCCGGGATAATTTCTGATGGCTGTCGCAGCAATGATGGCGATTCAGATAGTATGTCATCTGCTTGTCATGAGCGAAAGATTATTGTTGCAAACATGCTGCCTTTGCATGCTCAAAGGGATACAACAGCTGAAAAGTGGTGCTTTAGTTTGGACGAGGATTCACTTTTATTACAACTGAAGGATGGGTTTTCACCTG

***SlTPS5* (Solyc05g005750)**

ctgaaagtttcaagcatagatttcaaattccggcagtgcgtgtttaacaacgtagaccaaattttattttataatcATGCCATCAAGATCTTGTGCTAATCTTTTGGACATGGCATCTGGAGATATACTAGATATACCTCAGACACCTAGAGGTCTTCCACGTGTGATGACAGTTCCTGGAATTATCGCAGATGGTGATTCTGATGGTATGTCATCCTCATGTCGAGAGCGAAAAATTGTTGTAGCAAACATGCTGCCTTTGCATGCTCAAAGGGATACAACAGCTAAAAAGTGGTTATTCAGTTTGGATAAGGATTCTCTTTTATTGCAATTGAAGGATGGGTTTTCTCCTGAAACTGAGGTTGTCTATGTTGGTTCTC

***SlTPS6* (Solyc07g006500)**

gtgtttgctctgtttatggtgtaaagttttgaactttgtgattaaaaagtgttgatttttgatattcaagttaatttttttttgtgtgttgtgattATGTTGTCAAGATCTTGTTTCAATCTGCTTAATCTTGATGACTGTTCTGTTACTGATCGGGCTCGAATCCCAAAGTTGATGAATGTTCCAGGGATTATAACAGATTTTGGTGGAGGAGGAGGAGAAGAAGAGAAGGGGGAAGTTTCACCTGGTGTGAAAAATGGGAGTAGAAGGATCATTGTAGCAAATCAGTTACCAGTGAAAGCTTTTTGTAAAGATGAAAAAGAGGGGAAAAAATGGTGTTTTGAATGGGACAGATATGCTTTAGATACATTGATTTTGCAGCTGAAAGA

***SlTPS7* (Solyc07g055300)**

taaagatcgagtctttcactgaaaaagatccaagatttggtgctttttgaatcctagagaaagttgaaatacccaaattagaggaagtcaagaatctttgttagaATGATGTCTAGATCGTATACAAATCTTTTGGATTTGGCATCTGGGAATTTTCCTGTAATGGGAAGAGAGAGAGATAGGCGACGGATGTCGCGGGTAATGACAGTGCCTGGGAGTATATGTGAACTCGATGATGACCAGGCTGTTAGTGTTTCTTCTGATAATCAATCTTCACTTGCCGGTGATCGGATGATTGTTGTGGCGAATCAGTTGCCATTGAAAGCGAAAAGGAGACCGGATAATAAGGGCTGGAGTTTTAGTTGGAATGAGGATTCT

***SlTPS8* (Solyc07g062140)**

tcagaatctaatccttcatcgctcctcttcaatttcagactagcagaaacactttccgcacggtgtttcagaatctaaacaatcatcgatctctgctttaatttcaggtggtcttgttctagacagATGCCAGGGAACAAGTATACCGGCAACCAAGCGGTTGCTAGCACTCGATTGGAGAGGCTATTGAGAGAAAGAGAGCTTAGGAAAAGTAGCAAAGTTTCTCACTTTCCAAATGAATCTACTGATAACAATAGGGGAAACGAGCTCTCTGACCATGATTTTCGCCAAGGAGAAGCTGATAATGGAGGAGTTTCATATGTCGAACAGTACCTCGAAGGA

***SlTPS9* (Solyc08g076650)**

catatgccatcagatttatcctctagggtggtggctagacttctgaaaaataaaagagggatagttcattagctaaaaaaATGGTTTCAAGGTCATATTCCAACTTACTAGATCTTATTTCTGATGATTCGCCAACATTTGGCCGAGGGGGTAGAAAGCTTTCGAGGGTAGCAACTGTGGCTGGGGTTTTATCTGAGCTTGATGATGAAAGTAGAAGTAATGCTTCTGATGCTCCATCATCAGTTACTCAAGAGAGGATGATAATTGTGGGGAATCAACTCCCTCTTAGAGCTCATAGGAGACAAGATGGTGAGGAAGGATGGAACTTTAGTTGGGATGAGGATTCCCTTCTTTTACAGCT

***SlTPS10* (Solyc10g007950)**

caaaggggtttctgataattgaatctctgaggtaattaagaactacctgattaaggggttgaggtggcattggaaagacactgaagaatattagtaaaaATGATTTCGAGATCGTATACCAACCTTTTGGATTTGGCTTCTGGGAATTTTCCGACAATGGGAAGAGATAGGGACCGGAGACGGATGCCAAGGGTGATGACACTACCTGGGAGTATATGTGAGATGGATGATGACCAGGCTCATAGTGTTTCATCTGAGAATCCGTCTTCGCTGGCTGGTGATCGGATGATTGTGGTGGCAAATCTGTTGCCCCTGAAAGCAAAAAGGAGACCAGACAATAAAGGCTGGAGCTTTAATTGGAATGAGGACTCGTTACTTTTGAGACTT

***SlTPP2* (Solyc03g083960)**

agggttggttaaaagtgtgtgaaacaatttaagagcagagttactcaaaccattgtcaactgcttgcatagttttaaacttattttctgccataaaaaagatactaggcacaATGGACCTGAAATCAAATACATCCCCTGTTGTCACCGATCCTGCCCCACTGACTCCGTCCAGATTGGGCATCCACTCAGCTTTGATGCCATACTCTCCGGCTTTGCCAACTTTCTCCCCCACTCTCTTCCTTACTATCCCAAGGAAGAAGCCTGGAATTTTAGATGATGTTAGATCAAATACTTGGTTGGTGGATGCCATGAAATCTTCATCTCCTA

***SlTPP3* (Solyc04g054930)**

tactctgaattctcattctcattcccattttcctttacataaaacagagtgaaaacagaaccaaacacacactgccttcaatattccacctcaaatatactataatttaggagtgacaATGACTCAGCAGAATGTGGTAGTATCTGATCCCAAATCCGGTATTAATCTGACAATACCGGTGAAGGTACCGGTATCGAACTCGTCGGCGTTGTTCACGACGGCAGCTCAGAAGCCACCGCCGGGGCCGGGGAGTTGTATCACTATTTCAAGAAAGACACTTCTTGAAATTAATGGGAATAATAGTGCTAGAATCAATTCTTGGGTTGAATCAATGAGAGCTTCCTCACCTACTCATCACAA

***SlTPP4* (Solyc04g072920)**

ttcccactcaatcaaattcttcgaatttgctctgtttttcaacaatcagttgtatagcaaaaATGACTAATCAGAATGTGATAATTTCTGACCCGAGATCAGGATTCGACTCTTCACTCTTATCATTGTCCCCTGCCGTTCCCGGTCCACTACCGCAGCCGGGAAGATTCATCGCTGTTCCAGCGAAGAGATCATTCAAAAATATTGATTCTACTGATGCAGCTAGAATCACTGCTTTACTTGATTCCATGAGAGCTTCTTCTCCAACTCGAAGATCCTCAGAAACTGAAAATCTCAACTCCTGGATTGTTCATCATCCATCAGCTCTGAATATGTTCGATGAA

***SlTPP8* (Solyc08g079060)**

tcattgataaaagggttggttaaaaattcgctactcccaaacatccactataaagtatcaactgcatacctatcaattgcatagtcaaactctgccgaatccatagtgttgcaATGGACTTGAATTCGGCACAAGCTTCTCCAGTTCTTACTGATCCTTCACCGTTGAACAAGTCCAGATTGGGAATCCATTCTAGTTTATTTCCTTATTCACAATCTGGGCCTTCATTCTCCACTAGTGTGCTATCAATTCCAAGAAAAAAGCCAGCGAAGCTTGATGATGTTCGATCCAATGGTTGGCTTGATGCAATGAAGTCGTCTTCACCT

***SlTRE1* (Solyc08g082860)**

cacataaattagttatggcatcagtattgaactctttaacttgttatacaacATGGGTAAAGCTATAATTTTTATGATTTTTACAATGTCTATGAATATGATTAAAGCCGAAACTTGCAAATCCGTTGATAAGGGTCCTGTAATCCCAACAACCCCTTTAGTGATTTTTCTTGAAAAAGTTCAAGAAGTTGCTCTTCAAACTTATGGCCATAAAAGGGTTGATGCTAAACTGTTTGTTGATATGTCACTGAGAAAGAATCTTTCAGAAACAATTGAAGCTTTTAATAAGCTTCCAAGAATTGTGAATGGTTCAGTATCAAAGAGTGATTTGGATGGTTTTATTGGTAGT

**Supplementary Table S1.** Primers used in this study for different purposes

| Primers | Sequences (5’-3’) | Size (bp) |
| --- | --- | --- |
| ***VIGS*** | | |
| SlTPP2-VIGS-F | AGGGTTGGTTAAAAGTGTGT | 329 |
| SlTPP2-VIGS-R | TAGGAGATGAAGATTTCATG |
| SlTPP3-VIGS-F | TACTCTGAATTCTCATTCTC | 360 |
| SlTPP3-VIGS-R | TTGTGATGAGTAGGTGAGGA |
| SlTPP4-VIGS-F | TTCCCACTCAATCAAATTCT | 344 |
| SlTPP4-VIGS-R | TTCATCGAACATATTCAGAG |
| SlTPP8-VIGS-F | TCATTGATAAAAGGGTTGGT | 326 |
| SlTPP8-VIGS-R | AGGTGAAGACGACTTCATTG |
| SlTPS1-VIGS-F | ATGTTAGGAGCTTACTATAG | 368 |
| SlTPS1-VIGS-R | TGAATTCTCATCCCAGCTGA |
| SlTPS3-VIGS-F | GTGATGTATGTGGATTGAGT | 378 |
| SlTPS3-VIGS-R | AACGAATCCTCATCCCAACT |
| SlTPS4-VIGS-F | ACACACTTTCCTTTGGTTTC | 371 |
| SlTPS4-VIGS-R | CAGGTGAAAACCCATCCTTC |
| SlTPS5-VIGS-F | CTGAAAGTTTCAAGCATAGA | 380 |
| SlTPS5-VIGS-R | GAGAACCAACATAGACAACC |
| SlTPS6-VIGS-F | GTGTTTGCTCTGTTTATGGT | 389 |
| SlTPS6-VIGS-R | TCTTTCAGCTGCAAAATCAA |
| SlTPS7-VIGS-F | TAAAGATCGAGTCTTTCACT | 378 |
| SlTPS7-VIGS-R | AGAATCCTCATTCCAACTAA |
| SlTPS8-VIGS-F | TCAGAATCTAATCCTTCATC | 342 |
| SlTPS8-VIGS-R | TCCTTCGAGGTACTGTTCGA |
| SlTPS9-VIGS-F | CATATGCCATCAGATTTATC | 361 |
| SlTPS9-VIGS-R | AGCTGTAAAAGAAGGGAATC |
| SlTPS10-VIGS-F | CAAAGGGGTTTCTGATAATT | 380 |
| SlTPS10-VIGS-R | AAGTCTCAAAAGTAACGAGT |
| SlTRE1-VIGS-F | CACATAAATTAGTTATGGCA | 349 |
| SlTRE1-VIGS-R | ACTACCAATAAAACCATCCA |
| ***qRT-PCR*** | | |
| SlTPP2-qRT-F | AGGAGATGATCGTACAGATGAA | 119 |
| SlTPP2-qRT-R | GATCCCTCAGAGAGTAGAATGC |
| SlTPP3-qRT-F | CTGAGGTTATGGTGTTTCTACG | 110 |
| SlTPP3-qRT-R | GTTCCTTAGAGATGCTTTCATC |
| SlTPP4-qRT-F | AGGCTTTGGAATATTAGTGTCC | 106 |
| SlTPP4-qRT-R | CCACTCCACCAAACGATTCAAA |
| SlTPP8-qRT-F | AAGAGAGCAATGCATCCTTT | 93 |
| SlTPP8-qRT-R | CATCATCTTCAACTTCCATCGT |
| SlTPS1-qRT-F | GCTCCAGCAGCCGAAGTCTT | 100 |
| SlTPS1-qRT-R | CCTGCATCAGCCTAACTATCTC |
| SlTPS3-qRT-F | TCTTCAAGCACGGAAGTGTATG | 82 |
| SlTPS3-qRT-R | CTTCTGCGGTGTCATCAAGATA |
| SlTPS4-qRT-F | AGAAGCCAAGCAAAGCCAAG | 80 |
| SlTPS4-qRT-R | AGAAGCATTAGCAAGACCTCCA |
| SlTPS5-qRT-F | AAAGCCAAGCAAAGCCAAGTA | 83 |
| SlTPS5-qRT-R | GACAGGACGCATTACCAAGAC |
| SlTPS6-qRT-F | TTCAGCAGCTTCTACAGCAATG | 80 |
| SlTPS6-qRT-R | CTAGGGAGAGATCTTCGACAGA |
| SlTPS7-qRT-F | ACACATCTGAGGTGGTGCTTAT | 89 |
| SlTPS7-qRT-R | GGAGTCCGCAGGTTCATCGT |
| SlTPS8-qRT-F | GTGTCATGGAATGTGCTGGAT | 96 |
| SlTPS8-qRT-R | TGGCGTACTGAGCAGATACC |
| SlTPS9-qRT-F | GGCCAGAAACCAAGCAAGG | 87 |
| SlTPS9-qRT-R | CTCAGAAGCAGAAGCCAGG |
| SlTPS10-qRT-F | TCGGTGATGCTTTATCCAGAAA | 113 |
| SlTPS10-qRT-R | AACCTCTGAAGTGTCGTCCAA |
| SlTRE1-qRT-F | ACGGGTTTCGGATGGTCAAA | 80 |
| SlTRE1-qRT-R | GCAGTCATTCTTCAAATCTTCG |
| SlPR1b-qRT-F | TTTCCCTTTTGATGTTGCT | 96 |
| SlPR1b-qRT-R | TGGAAACAAGAAGATGCAGT |
| SlPRP2-qRT-F | CGATCTAAATTGATTTCATAGTACG | 116 |
| SlPRP2-qRT-R | TCGTGAAGGATATACAAAATACA |
| SlLapA-qRT-F | GGGACTAATGATGTTTGGAA | 109 |
| SlLapA-qRT-R | GTGGCAATTTTATTTAGGCA |
| SlPIN2-qRT-F | CATCTTCTGGATTGCCCA | 106 |
| SlPIN2-qRT-R | ACACACAACTTGATGCCCAC |
| BcActin-qRT-F | CGTCACTACCTTCAACTCCATC | 107 |
| BcActin-qRT-R | CGGAGATACCTGGGTACATAGT |
| SlActin-qRT-F | CCAGGTATTGCTGATAGAATGAG | 113 |
| SlActin-qRT-R | GAGCCTCCAATCCAGACAC |

**Supplementary Figure S1 | Silencing of *SlTPS7* or *SlTPS8* led to inhibition of vegetable growth in tomato plants.** Ten-day-old tomato plants were infiltrated with agrobacteria carrying TRV-SlTPS7, TRV-SlTS8 or TRV-GUS constructs and the photos were taken and the plant heights were recorded at 4 weeks after agroinfiltration. At least 10 TRV-SlTPS7, TRV-SlTPS8- or TRV-GUS-infiltrated plants were used in each of three independent experiments and similar results were obtained in independent experiments (A). Data presented in (B) are the means ± SD from three independent experiments and * above the columns indicate significant differences at *p*<0.05 level between the TRV-SlTPS7/SlTPS8-infiltrated plants and TRV-GUS-infiltrated plants.

**
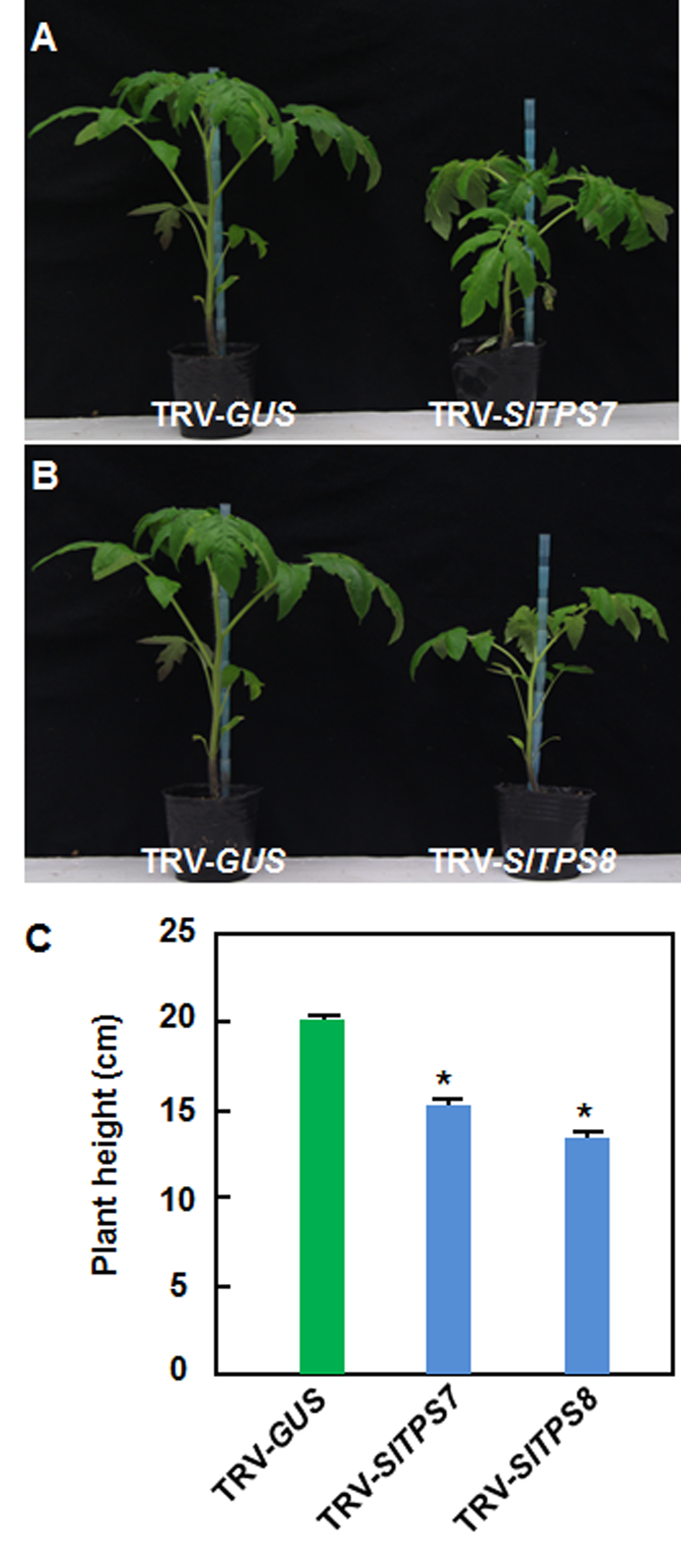
**
